# Comparative study of neutralizing antibodies titers in response to different types of COVID-19 vaccines among a group of egyptian healthcare workers

**DOI:** 10.1186/s12985-024-02546-0

**Published:** 2024-11-05

**Authors:** Sara Maher, Nihal M. El Assaly, Doaa Mamdouh Aly, Shimaa Atta, Asmaa Mohamed Fteah, Hala Badawi, Manal Youssef Zahran, Manal Kamel

**Affiliations:** 1 Immunology Department, Theodor Bilharz Research Institute, Giza, Egypt; 2 Clinical Chemistry Department, Theodor Bilharz Research Institute, Giza, Egypt; 3 Microbiology Department, Theodor Bilharz Research Institute, Giza, Egypt; 4 Hematology Department, Theodor Bilharz Research Institute, Giza, Egypt

**Keywords:** COVID-19, Neutralizing antibodies, BNT162b2, Sinovac, ChAdOx1

## Abstract

**Background:**

Defining the protective thresholds against the severe-acute-respiratory-syndrome-related corona virus-2 pandemic is a crucial challenge. To reduce the risks of severe disease, hospitalization, and death, various COVID-19 vaccines have been rapidly developed.

**Aim of the work:**

This study aimed to assess the impact of three common COVID-19 vaccine types; two mRNA COVID-19 vaccines: (Pfizer/BioNTech’s BNT162b2 and Moderna’s mRNA-1273), one adenoviral vector vaccine: Oxford/AstraZeneca’s ChAdOx1, and one inactivated vaccine (Sinovac Biotech/China’s Sinovac) on the level of neutralizing antibodies, considering factors such as vaccine type, demographic characteristics, and hybrid immunity. We conducted a direct comparative analysis involving 300 healthcare workers, both with and without prior SARS-CoV-2 infection (B.1, C.36.3, and AY.32 (Delta) variants). Neutralizing antibodies levels were measured at baseline (before vaccination), before the second dose, and six months after the second dose.

**Results:**

The results showed a significant increase in neutralizing antibodies levels after complete vaccination with all vaccine types. Among healthcare workers, those vaccinated with mRNA vaccines (Moderna or Pfizer) exhibited the highest neutralizing antibodies titers, followed by AstraZeneca, and finally Sinovac with the lowest titer. On studying the effect of previous COVID-19 infection after vaccination, no significant difference in neutralizing antibodies levels was observed between healthcare workers vaccinated with mRNA or AstraZeneca vaccines, both with prior COVID-19 infection, following the first and six months after the second dose.

**Conclusion:**

These findings suggest that individuals with prior COVID-19 may only require a single dose of mRNA or AstraZeneca vaccines to achieve a similar level of immunization as those without prior COVID-19 who completed the vaccination program.

**Highlights:**

- There is a significant increase in neutralizing antibodies levels after complete vaccination against COVID-19
- Vaccination with mRNA vaccines exhibits the highest neutralizing antibodies titers.
- Vaccination with Sinovac exhibits the lowest neutralizing antibodies titers.

**Graphical abstract:**

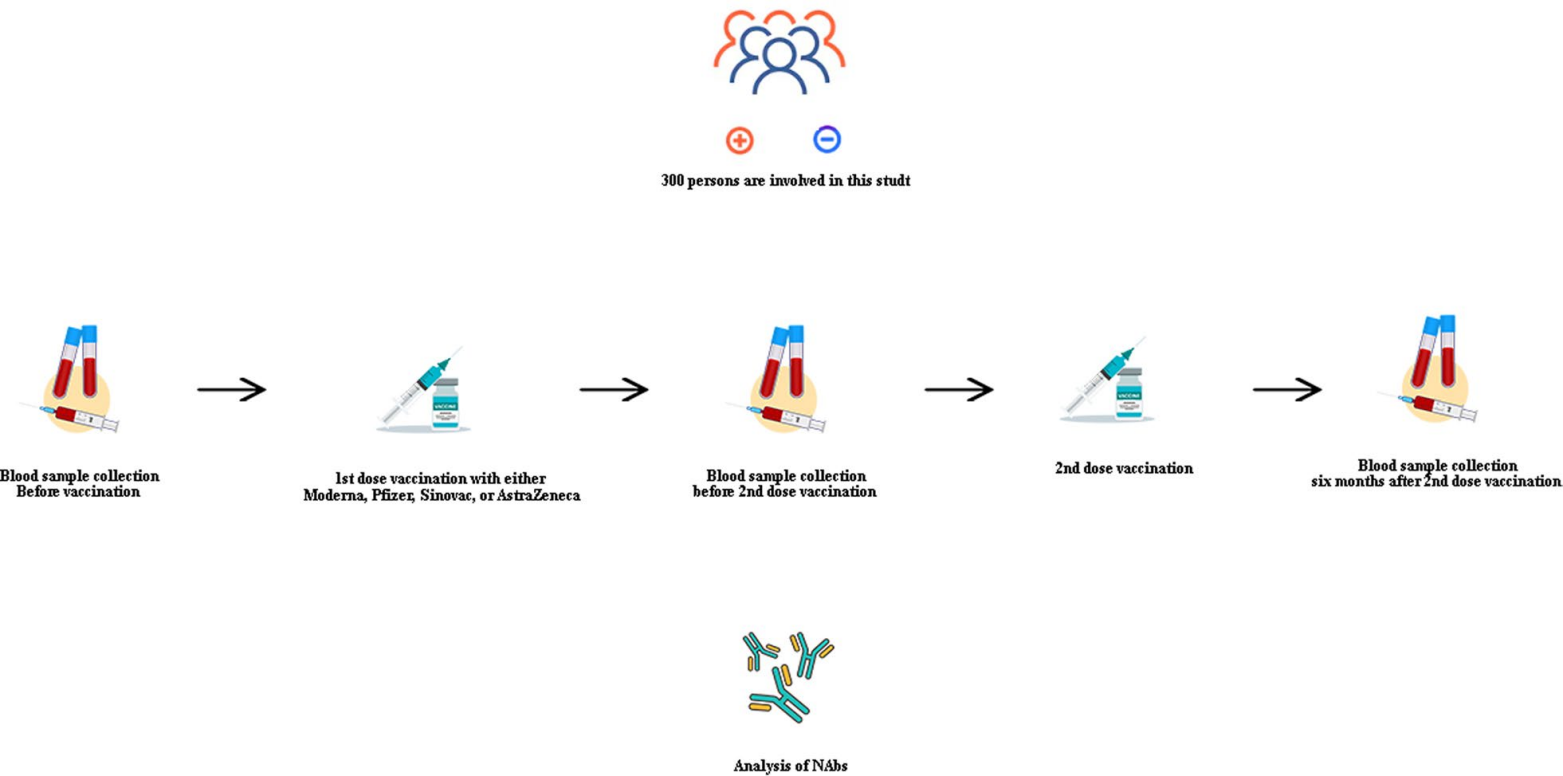

**Supplementary Information:**

The online version contains supplementary material available at 10.1186/s12985-024-02546-0.

## Introduction

By the end of 2019, a novel coronavirus disease, COVID-19, emerged in the city of Wuhan, China, causing a sudden outbreak of atypical pneumonia. This outbreak has since turned into a pandemic that continues to spread globally [1]. The COVID-19 crisis represents a moment of rupture as well as continuity for vulnerable labor in Egypt and worldwide. On March 11th, 2020, the World Health Organization (WHO) declared the disease a pandemic, citing its rapid spread and global scale [2], resulting in very high morbidity and mortality rates [3].

In Egypt, from January 2020 to June 2023, there were 516,023 confirmed cases of COVID-19, with 24,830 deaths reported to the WHO. As of May 20, 2023, a total of 112,673,535 different vaccine doses have been administered in Egypt [4]. The outcome of a SARS-CoV-2 infection can vary widely and depends on demographic factors, and underlying conditions such as diabetes, obesity, and Immunocompromised [5]. Currently, it is still uncertain whether a previous SARS-CoV-2 infection can provide protection against subsequent infection [6]. Due to this uncertainty, some clinical trials of COVID-19 vaccines excluded participants who had a previous infection [7]. However, the US Food and Drug Administration (FDA) has recommended that these individuals could be included in vaccine trials [8].

Vaccination is the most effective and promising public policy tool for remission of COVID-19 infections, and mortality [9]. Vaccine development normally takes years or decades, but the urgency of the pandemic led to unprecedented efforts from all countries around the world. This resulted in accelerated progress in the development and subsequent approval of COVID-19 vaccines within a short timeframe [10].

Currently, one COVID-19 vaccine, BNT162b2 (Pfizer), has received full approval from the US FDA. The other vaccines, including mRNA-1273 (Moderna), ChAdOx1 nCoV-19 (Oxford/AstraZeneca, Adenovirus-based), and CoronaVac (Sinovac Biotech, Whole-cell in-activated virus), have been approved for Emergency Use Authorization (EUA) by the FDA since December 2020 [11]. In clinical trials, the BNT162b2 vaccine showed 95% protection against COVID-19 following two doses [12].

The humoral immune response and production of specific antibodies against pathogens like SARS-CoV-2. One of the virus’s structural proteins is the spike protein is the most antigenic one, and most serological tests detect antibodies against it [13]**.** Those antibodies that are capable of neutralizing the antigen’s critical functions are considered neutralizing antibodies (NAbs). In the case of SARS-CoV-2, antibodies that neutralize the virus’s spike protein are the most potent. However, the efficacy of NAbs can be affected by the mutations that arise spontaneously in the virus [14]. In the recent COVID-19 infection, NAbs can be used as both therapeutic and diagnostic tools for immune surveillance and it is playing a key role in the context of the global vaccination strategy and for plasma therapy [3]. Compared with SARS-CoV-2 Ig M and Ig G antibody assays, detecting NAbs reliably measure the real protective immunity of antibodies [15].

There is strong evidence that the presence of neutralizing antibodies is highly predictive of protective immunity [16]. Concentrations and titers of neutralizing antibodies were inversely correlated with the risk of symptomatic COVID-19 infection and directly related to the vaccine efficacy [17].

Despite the role of the COVID-19 vaccination in reducing the virus morbidity and mortality, many studies reported the occurrence of vaccine-induced immune thrombocytopenia and cerebral venous sinus thrombosis after vaccination with the COVID-19 vaccines. Nevertheless, these complications are very rare, yet are potentially fatal [18]**.** That’s why the correlation between the NAbs and platelet count and D-dimer levels should be investigated.

The current gold standard method to assess NAbs is the conventional virus neutralization test (that relies on cell-culture-based infection), which requires a biosafety level 3 laboratory to manipulate the live pathogen, high workload, skillful technicians, and expensive installations, and they exhibit limited capacity [19]. The use of a SARS-CoV-2 blocking ELISA kit that surrogate virus neutralization test (sVNT), depending on antibody-mediated blockage of the interaction between the RBD and ACE2 receptor protein, has been found to be an efficient alternative [20]. This test achieves 95–100% sensitivity and 99.93% specificity [21, 22]. Remarkably, Garcia-Beltran et al*.,* 2021 found that neutralization titer, and potency were directly associated with disease severity and predicted survival [23]. NAbs immune memory to COVID-19 infection could be generated by natural immunity through a previous infection, vaccination, or hybrid immunity [24]. Hybrid immunity is the combination of vaccine-induced immunity and infection-induced immunity. Recent studies revealed that hybrid immunity results in more strong protection against COVID-19 than either previous infection immunity alone or vaccine-induced immunity alone [25, 26].

In this study, we aimed to compare the presence and level of neutralizing antibodies in HCWs who have and have not been previously infected with SARS-CoV-2 after receiving COVID-19 vaccines. The objective was to gain a better understanding of the differences in immunogenicity between various COVID-19 vaccines and to determine the impact of natural infection on NAbs level.

## Materials and methods

### Ethical approval

This study was approved by the Research Ethics Committee (REC) at Theodor Bilharz Research Institute (TBRI) (#PT 594, 2021). The human subjects in this study were enrolled according to REC-TBRI’s ethical standards and the 1964 Helsinki Declaration. A signed consent form was obtained from each participant before sample collection. This study is observational; it is not a clinical trial.

### Study population

In this study which was carried out from May 2021 to December 2022, we investigated neutralizing capacity by means of blocking ELISA in 300 healthcare workers (HCWs) from the hospital who had been vaccinated with COVID-19 vaccines. They were divided into 4 groups according to the type of vaccine given: AsterZenca, Sinovac, Pfizer-BioNTech (BNT162b2) and Moderna.

Inclusion criteria

- Healthcare workers (HCWs) in our institute aged from 25—70 years old.
- Individuals who denied having recent history of COVID-19 and were confirmed to be SARS CoV-2 IgM seronegative before vaccination by Immunoglobulin M (IgM) Rapid Test (Rightsign, China).

Exclusion criteria

- Pregnancy
- History of recent COVID-19 infection or positive IgM by rapid test before vaccination, as this means that the person is having an ongoing COVID-19 infection. According to the guidelines, the person with theaes criteria should be excluded from the vaccination program until they become SARS CoV-2 IgM seronegative via IgM Rapid Test (Rightsign, China).
- Individuals undergoing treatment with immunosuppressive drugs.

### Sample collection

Nasopharyngeal swabs for the detection of antigens as well as serum antibodies rapid tests were performed for 350 HCWs to investigate the COVID-19 current infection. Positive antigen rapid tests or IgM antibody subjects were excluded from this study. Peripheral blood was obtained from all individuals by venous puncture in vacutainer tubes into two parts, one for the routine test (complete blood picture (CBC), D-dimer, C reactive protein, Ferritin), the other part for serum collection where it was allowed to clot for 1 h at room temperature before centrifugation at 1300 rpm for 5 min. The supernatant was then stored at -20℃ till the time of the assay. Serum samples for detection of NAbs were obtained four times for each participant in the study:

1. Baseline sample: before start of vaccination scheduale.
2. Second sample: five days after the 1st dose of vaccination of either type.
3. Third sample: before the second dose of vaccines, three months after AstraZeneca 1st dose, and 21 days after Sinovac or mRNA vaccines 1st dose.
4. Fourth sample: six months after the 2nd dose of vaccination for all types.

### Measurement of SARS CoV-2 neutralizing antibodies and Rapid test methodology

Neutralizing antibodies assessment against SARS-CoV-2 in the collected serum samples was performed with the SARS-CoV-2 Neutralization Antibody Detection Kit (Elabscience Biotechnology Co., USA)—Catalog No: E-EL-E608 Product size: 24T/96T/96T*5, which is a competitive Enzyme-Linked Immunosorbent Assay (ELISA) according to the manufacturer’s instructions. First, the SARS CoV-2 neutralization Antibody in the serum samples or standards/controls competes with a fixed amount of recombinant human ACE2 on the solid phase supporter for sites on the Horseradish peroxidase (HRP) conjugated recombinant SARS CoV-2 RBD fragment (HRP-RBD). After 37℃ incubation for 30 min, the unbound HRP-RBD as well as any HRP-RBD bound to non-neutralization antibody was captured on the plate and eventually formed the ACE2-RBD-HRP complex, while the circulating neutralization antibodies HRP-RBD complexes remain in the supernatant and are removed during washing. Then a tetramethylbenzidine (TMB) substrate solution was added to each well. The enzyme–substrate reaction was terminated by the addition of stop solution (H_2_SO_4_) and the color change was measured spectrophotometrically at a wavelength of 450 nm ± 2 nm. The concentration of SARS-CoV-2 Neutralization Antibody in the samples is then determined by comparing the OD of the samples to the standard curve.

According to the manufacturer’s findings, a concentration less than or equal 16 ng/ml indicating seronegative subjects and positive cases have levels from 45 to 500 ng/ml.

The SARS-Cov-2 IgM and IgG were detected using the RightSign COVID-19 IgG/IgM Rapid Test Cassette. The kit uses the lateral flow immunochromtographic assay for the detection of SARS-CoV-2 antibodies in venous whole blood. This test uses anti-human IgM antibody (test line IgM), anti-human IgG (test line IgG) and goat anti and goat anti-mouse IgG (control line C) immobilized on a nitrocellulose strip. The conjugate pad contains recombinant SARS-CoV-2 antigen (Spike protein RBD domain main antigens of SARS-CoV-2) conjugated with colloid gold. During testing, the specimen binds with SARS-CoV-2 antigen- conjugated gold colloid coated particles in the test cassette. When a specimen followed by assay buffer is added to the sample well, IgM &/or IgG antibodies if present, will bind to COVID-19 conjugates making an antigen–antibody complex. This complex migrates through nitrocellulose membrane by soft capillary action. When the complex meets the line of the corresponding immobilized antibody (anti-human IgM &/or anti-human IgG) the complex is trapped forming a colored line which indicates a reactive test result. Absence of a colored line in the test region indicates a nonreactive test result. The kit is FDA approved with 100% specificity and 93.3% sensitivity [27].

### Statistical analysis

Data were coded and entered using the statistical package for the Social Sciences (SPSS) version 28 (IBM Corp., Armonk, NY, USA). Data was summarized using mean, standard deviation, median, minimum, and maximum in quantitative data and using frequency (count) and relative frequency (percentage) for categorical data. Comparisons between quantitative variables were done using the non-parametric Mann–Whitney test for comparing two groups or Kruskal–Wallis for three or more, followed by Dunn’s multiple comparisons. For comparison of serial measurements within each patient the non-parametric Friedman test and Wilcoxon signed rank test were used *(Chan, 2003a)*. For comparing categorical data between-group proportions, a Chi-square (χ2) test was performed. The Fisher Exact test was used instead when the expected frequency is less than 5 *(Chan, 2003b)*. Correlations between quantitative variables were done using the Spearman correlation coefficient *(Chan, 2003c).* The statistical significance level was taken at *p*-values < 0.05.

## Results

### Description of Study Groups and routine laboratory investigation results

Following antigen and antibody rapid screening tests for 350 nasopharyngeal swabs as well as serum samples from TBRI-HCWs, 300 samples showed IgM and antigen negative results revealing no recent COVID-19 infection, while 50 samples showed IgM and antigen positive results denoting presence of recent COVID-19 infection and they were excluded from the study. History of old COVID-19 infection was investigated by testing IgG levels with rapid test method. According to old history of infection the 300 participants were sub-grouped into with or without history of infection (figure S1 (in ESI file)).

NAbs titer was analyzed for those 300 cases before and following the vaccination with different COVID-19 vaccines including AstraZeneca (*n* = 105), Sinovac (*n* = 84), and mRNA vaccines (Moderna & Pfizer, *n* = 111). Table 1 and figure S2 (in Electronic Supplementary Information (ESI)) summarizes the population’s general characteristics, age, and gender.

**Table 1:** Demographic characteristics of included subjects

		AstraZeneca (*n* = 105)		Sinovac (*n* = 84)		Moderna and Pfizer (*n* = 111)	
		n	%	n	%	n	%
History of COVID-19 infection^*^	Yes	42	40	36	42.8	50	45
	No	63	60	48	57.1	66	59.4
Sex^*^	Male	40	38.1	34	40.4	46	41.4
	Female	65	61.9	50	60.2	65	58.5
Age^**^		48.00 (25–70)		46.0 (20–68)		40.0 (29–64)	

^*^Data are represented as a number (percent)

^**^Data are presented as median (interquartile range)

Regarding the D-dimer and PLT, NAbs titer before vaccination was negatively correlated with D-dimer level 5 days after vaccination with AstraZeneca (*r* = −0.216, 0 = 0.048) as well as 6 months following vaccination with mRNA vaccines (*r* = 0.615, *p* < 0.001). In the Sinovac vaccinated group, no significant results were obtained. In all groups, NAbs were positively correlated with PLT before 1st dose of vaccination (*r* = 0.232, *p* = 0.006) as well as 5 days following the 1st dose and 6 months after the 2nd dose (*r* = 0.269, *p* = 0.001 and *r* = 0.201, *p* = 0.018 respectively) (tables S1, S2, S3 and figures S3, S4, S5, S6 in ESI file). In correlation with other routine tests (CBC, C-reactive protein, and serum ferritin), no significant results were obtained in different types of vaccinated groups.

### Neutralizing antibodies response over time in all vaccinated HCWs

**Fig. 1:**
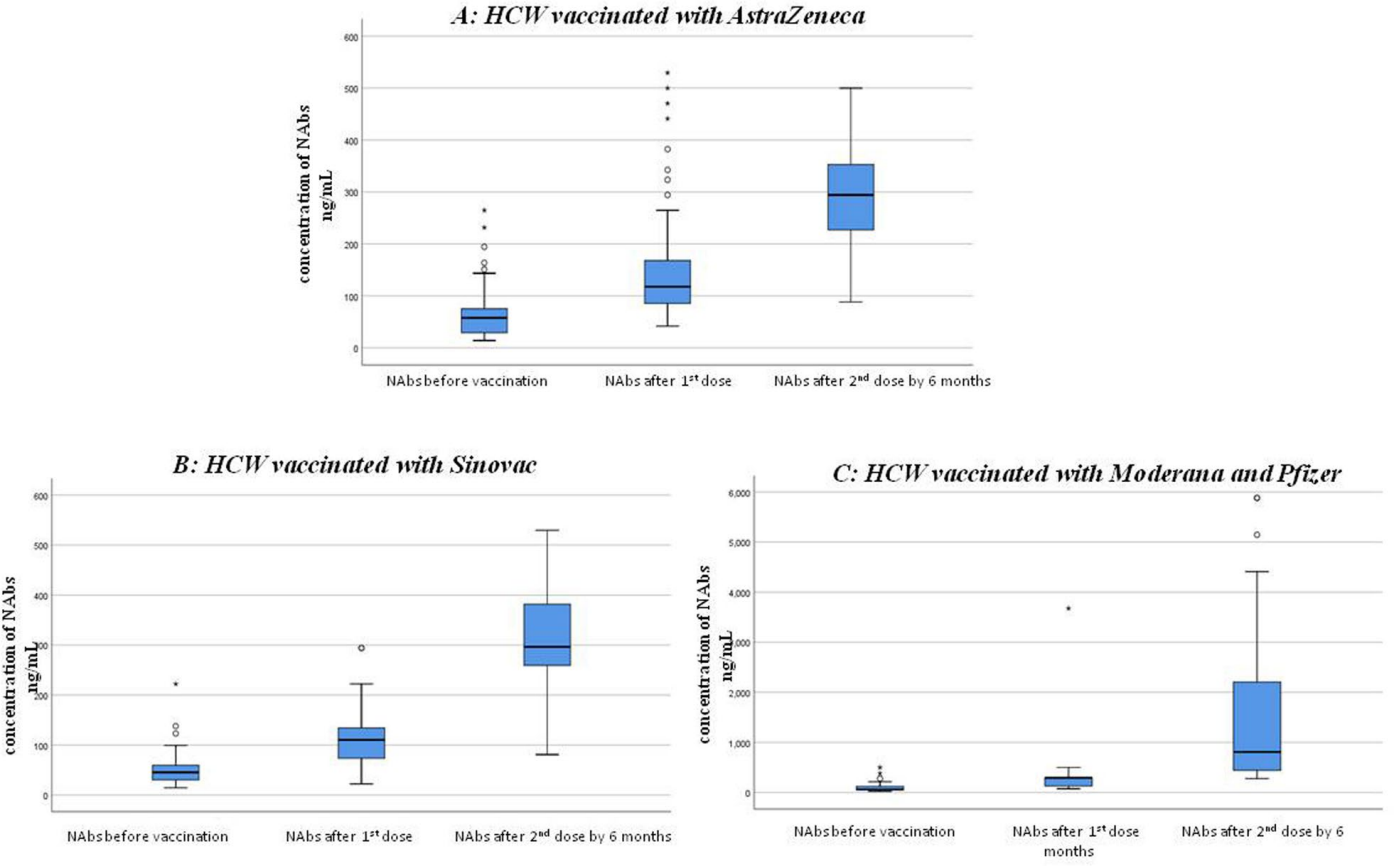
The graph shows the median (middle line) of NAbs level (ng/ml) before and after the 1st and the 2nd dose of vaccination with A (AstraZeneca), B (Sinovac) and C (Moderna & Pfizer) vaccines. We used independent samples Kruskal–Wallis H tests for statistical comparison, considering* p* < 0.05 as statistically significant the asterisk indicates outliers. *P value < 0.001 compared to before vaccination

The NAbs titer for each type of vaccine was evaluated over time between doses (Table 2 and Fig. 1). HCWs who received mRNA vaccines consistently exhibited the highest median NAbs levels throughout the study period (*p* < 0.001 for both doses). In HCWs vaccinated with AstraZeneca, there was a significant increase in the titer after the 1st and 2nd dose compared to the titer before vaccination (*p* < 0.001 for both doses). Similarly, Sinovac-vaccinated HCWs showed a significant increase in the median level of NAbs titer over time (*p* < 0.001 for both doses, respectively).

**Table 2:** Neutralizing antibody response over time in HCWs in each type of vaccine

	HCW vaccinated with AstraZeneca	*p*-value compared to before vaccination
Neutralizing antibodies Before vaccination	57.55 (14.07–264.71)	–
Neutralizing antibody after 90 days of the 1st dose	117.65 (41.67–529.41)	< 0.001
Neutralizing antibody after 6 months of 2nd dose by 6 months	294.12 (88.24–500)	< 0.001

	HCW vaccinated with Sinovac	P value compared to before vaccination
Neutralizing antibody Before vaccination	45.31 (14.62–222.22)	–
Neutralizing antibody after 21 days of the 1st dose	110.29 (22.40–294.12)	< 0.001
Neutralizing antibody after 6 months of 2nd dose by 6 months	296.30 (80.88–529.41)	< 0.001

	HCW vaccinated with Moderana and Pfizer	P value compared to before vaccination
Neutralizing antibody Before vaccination	59.38 (24.7–500)	–
Neutralizing antibody after 21 days of the 1st dose	294.12 (73.5–3675)	< 0.001
Neutralizing antibody after 6 months of 2nd dose by 6 months	808.50 (277.7–5880)	< 0.001

Data are presented as median (percentiles)

Moreover, we investigated the association between NAbs titers and prior COVID-19 infection in the HCWs before and after the vaccination, for different types of vaccines. Our results, presented in Table 3 and Fig. 1, indicated that HCWs who had a history of COVID-19 infection and received mRNA vaccines, AstraZeneca, or Sinovac had higher baseline NAbs levels compared to those who were vaccinated with the same vaccines but had not exposed to COVID-19 infection (*p* = 0.009, < 0.001, 0.033, respectively). We also found that HCWs who received the AstraZeneca vaccine and had a recent history of COVID-19 infection had significantly higher NAbs level after the first dose of the vaccine than those who received the same vaccine without a previous infection (*p* = *0.026*).

**Table 3:** Relation between history of COVID-19 infection and NAbs titer

	History of recent COVID-19 infection		
	Yes	No	*p value*
	Median (IQR)	Median(IQR)	
AstraZeneca			
NAbs Before vaccination	91.91 (41.67–194.44)	46.88 (14.07–264.71)	< 0.001^*^
NAbs after 1st dose	194.44 (75.37–441.18)	115.81 (41.67–529.41)	0.026^*^
NAbs after 2nd dose by 6 months	294.12 (117.65–382.35)	294.12 (88.24–500)	0.579
Sinovac			
NAbs Before vaccination	99.26 (29.17–222.22)	33.82 (14.62–67.71)	0.033^*^
NAbs after 1st dose	110.29 (56.99–294.12)	110.29 (22.4–294.12)	0.624
NAbs after 2nd dose by 6 months	382.35 (166.67–481.48)	295 (80.88–529.41)	0.278
Moderna and Pfizer			
NAbs Before vaccination	166.67 (46.88–500)	58.85 (24.78–82.72)	0.009^*^
NAbs after 1st dose	294.12(82.72–3675)	294.12 (73.53–500)	0.823
NAbs after 2nd dose by 6 months	735 (294.12–5145)	1470 (277.78–5880)	0.670

Data are presented as median (percentiles)

^*^Represents significant association

We also found that the NAbs titer following both the first and second doses of the COVID-19 vaccine was higher in HCWs who received two doses of the vaccine with a one-month interval between doses (mRNA and Sinovac), compared to those who received two doses of the AstraZeneca vaccine with a three-month interval between doses (*p* = *0.020, p* < *0.001*, respectively).

As regards, we compared the NAbs level in HCWs with and without history of COVID-19 infection following the first and six months after the second dose of vaccines (Table 4). We noticed that there was no significant changes in the NAbs level between the first and the second doses of AstraZeneca and mRNA vaccines in patients with history of infection (*p* = *0.182, p* = *0.05* respectively). However, in the Sinovac group, there was a significant difference in NAb level *(p* = *0.018).* In contrast, among HCWs with no history of COVID-19 infection, we observed a significant difference in NAbs levels between the first and second doses of all COVID-19 vaccines (*p* < *0.001*).

**Table 4:** Intra-group comparisons of NAbs level in vaccinated HCWs with and without prior COVID-19 infection

	NAbs after 1st dose	NAbs after 2nd dose by 6 months	*p-*value
	Median (IQR)	Median (IQR)	
History of recent COVID-19			
AstraZeneca	194.44 (75.37–44.18)	294.12 (117.65–382.35)	0.182
Sinovac	110.29 (56.99–294.12)	382.35 (166.67–481.48)	0.018^*^
Moderna& Pfizer	494.12 (123.72–3675)	635.00 (294.12–4145.00)	0.060
No history of recent COVID 19			
AstraZeneca	115.81 (41.67–529.41)	294.12 (88.24–500.00)	< 0.001^*^
Sinovac	110.29 (22.40–294.12)	295.21 (80.88- 529.41)	< 0.001^*^
Moderna& Pfizer	294.12 (73.53–500.00)	770.00 (277.78–3880.0)	< 0.001^*^

Data are presented as median (percentiles)

^*^Represents significant association

### Relation between demographic characteristics and the level of the NAbs immune response in all vaccinated HCWs

By the assessment of the correlation between age and the NAbs response in vaccinated HCWs, we found that the level of NAbs before and after vaccination with the 1st dose is negatively correlated with age on vaccination with AstraZeneca vaccine only with no significant difference on vaccination with Sinovac, Pfizer or Moderna vaccines (Table S4 in ESI file). Furthermore, for all types of vaccines, our study showed that there was no significant difference in The NAbs titer between male and female HCWs (Table S5 in ESI file).

## Discussion

Comparing the different types of COVID-19 vaccines in terms of the NAbs titers developed after vaccination is a crucial step in evaluating vaccines. In this study, we investigated the dynamics of NAbs titers in healthcare workers (HCWs) at our institution over a six-month period following the administration of different vaccine types: BNT162b2 by Pfizer/BioNTech, and mRNA-1273 by Moderna (mRNA COVID-19 vaccines), ChAdOx1 by Oxford/AstraZeneca (adenoviral vector vaccine), and Sinovac by Sinovac Biotech/China (inactivated vaccine). To the best of our knowledge, this is the first study in Egypt to investigate NAbs’ response following COVID-19 vaccination with these four available vaccines. Our results showed that the variation in the NAbs response to the SARS-CoV-2 spike protein after receiving the full vaccine dose depended on demographic characteristics. The response was lowest among older age groups for Pfizer and AstraZeneca vaccines, but not for the Sinovac vaccine. Similar to our findings, Collier et al. (2021) reported lower NAbs titers in participants aged above seventy, which could be attributed to the aging process leading to a decline in the production of memory cells that produce neutralizing antibodies [28]. In contrast to our results, Evans et al. (2022) found no significant correlation between age and neutralizing antibodies [29]. We also found no significant differences in NAbs levels based on sex. Similar to our findings, Nanda et al. (2023) reported that the levels of NAbs were independent of demographic factors such as sex [30].

Generally, we observed a significant increase in NAbs levels over a six-month period following the administration of two doses of all vaccine types. However, HCWs vaccinated with mRNA vaccines showed the highest median increase in NAbs levels over time. This indicates that COVID-19 vaccines have elevated the level of protection against infection, thereby reducing morbidity and mortality [31].

We found that HCWs vaccinated with mRNA vaccines (Moderna and Pfizer) had significantly higher NAbs titers after the first dose of the vaccine, and these titers remained higher after the second dose even at six months, compared to both the AstraZeneca and Sinovac vaccinated groups. The AstraZeneca vaccine exhibited intermediate NAbs levels, following the mRNA vaccines, while the Sinovac vaccine had the lowest NAbs titer. Similar to our findings, Adjobimey et al*.* (2022) reported in their study involving 365 individuals vaccinated with Moderna, Pfizer, AstraZeneca, and Sinopharm that Moderna and Pfizer vaccines elicited the highest concentrations of COVID-19-specific neutralizing antibodies compared to other vaccine types, followed by the AstraZeneca vaccine [32]. The Sinopharm vaccinated group had the lowest neutralizing antibody titer. Sinovac and Sinopharm both belong to the same family of inactivated virus vaccines. Moreover, van Gils et al*.* (2022) found that mRNA vaccines induced higher neutralizing antibody titers compared to adenovirus vaccines after one month of vaccination [33]. Strong evidence from several studies concludes that the protective immune response due to neutralizing antibodies elicited by a two-dose AstraZeneca vaccine is lower and wanes faster than that of mRNA vaccines [34], which is consistent with our results. These findings may be attributed to the fact that mRNA vaccines encode a codon-optimized synthetic version of the full-length spike glycoprotein (S) of the COVID-19 virus, which facilitates efficient expression. This protein plays a crucial role in the development of neutralizing antibodies that can block viral entry into cells and prevent viral replication [35, 36]. The level of neutralizing antibodies against the S1 protein of COVID-19 is strongly correlated with immunity and protection against the virus after vaccination [37, 38]. Multiple studies have shown that two-dose mRNA vaccines (Moderna and Pfizer) elicit high levels of neutralizing antibody immune response against the SARS-CoV-2 ancestral strain as well as variants such as the Alpha and Delta strains [39, 40].

To study the impact of hybrid immunity on NAbs levels following vaccination with different types of COVID-19 vaccines, we assessed the relationship between NAbs titers before and after vaccination in individuals with a history of previous COVID-19 infection. HCWs who had been previously infected with COVID-19 had significantly higher baseline neutralizing antibody titers across all four vaccine types compared to HCWs without a history of COVID-19 infection. Consistent with our results, Virk et al*.* (2023) found in their study involving 48 HCWs that neutralizing antibody values in HCWs previously infected with COVID-19 were six times higher than those without a history of infection, six months after vaccination [25]. This indicates that breakthrough infection can enhance the durability of the neutralizing antibody response to COVID-19. This could be explained by the presence of hybrid immunity in HCWs who had previous COVID-19 infection. Some B-memory cells in individuals with prior infection produce antibodies with higher potency and broad reactivity. After vaccination, these cells are triggered to mount a robust response composed of antibodies capable of neutralizing various COVID-19 variants [41, 42].

One of the main goals of our study was to investigate the impact of previous COVID-19 infections on the necessity of vaccine doses. We compared neutralizing antibody titers after the first dose and six months after the second dose in HCWs with and without prior COVID-19. We found no significant change in neutralizing antibody levels following the first and second doses of the AstraZeneca and Pfizer/Moderna vaccines in HCWs with previous COVID-19 infections. However, we observed a significant difference in neutralizing antibody levels between the first and second doses in the Sinovac group. Our findings suggest that a single dose of the Pfizer/Moderna or AstraZeneca vaccines may be sufficient to provide similar immunization in individuals with a previous history of COVID-19 infection for at least six months. These findings are consistent with recent research by Goel et al. (2021), Chia et al. (2021), and Ebinger et al. (2021) [43–45]. Given the large population and limited financial resources in our country, these studies are crucial for optimizing vaccine allocation and controlling expenses.

Our study had some limitations. The first limitation is that the number of participants was relatively small. Another limitation of our study is that we currently lack results on the follow-up of neutralizing antibody titers in the study groups. It is important to conduct further investigations to monitor NAbs levels at least 12 and 18 months after vaccination to assess immune response and determine the strategy for booster doses. As well, it is important to follow-up breakthrough infection during the study period. We also recommend regular monitoring of neutralizing antibody levels in elderly patients and studying the fluctuations in their immune response over time.

## Conclusion

Our study supports the idea that individuals with prior COVID-19 may only need a single dose of mRNA (Pfizer/Moderna) or the AstraZeneca vaccines to achieve an immunization level comparable to those without prior COVID-19 who have received a complete vaccination.

## Supplementary Information

Additional file 1.

## Data Availability

No datasets were generated or analysed during the current study.

## Abbreviations

CBC: Complete blood picture
COVID-19: Coronavirus disease 2019
ELISA: Enzyme Linked Immunosorbent Assay
EUA: Emergency Use Authorization
FDA: Food and drug administration
HCWs: Health care workers
HRP: Horse raddish peroxidase
IgG: Immunoglobulin G
IgM: Immunoglobulin M
NAbs: Neutralizing antibodies
RBD: Receptor binding domain
SARS-CoV-2: Severe-acute-respiratory-syndrome-related coronavirus 2
sVNT: Surrogate virus neutralization test
