# Supplementary material for: Comparative study of neutralizing antibodies titers in response to different types of COVID-19 vaccines among a group of egyptian healthcare workers
Source: Virol J. 2024 Nov 5;21:277. doi: 10.1186/s12985-024-02546-0 (PMC11539826; doi:10.1186/s12985-024-02546-0)
Supplement: Supplementary file 1 — Additional file 1. [file 12985_2024_2546_MOESM1_ESM.docx]

**Electronic Supplementary Information**

**Title**

Comparative Study of Neutralizing Antibodies Titers in Response to Different Types of COVID-19 Vaccines among Egyptian Healthcare Workers, With or Without Previous Infection.


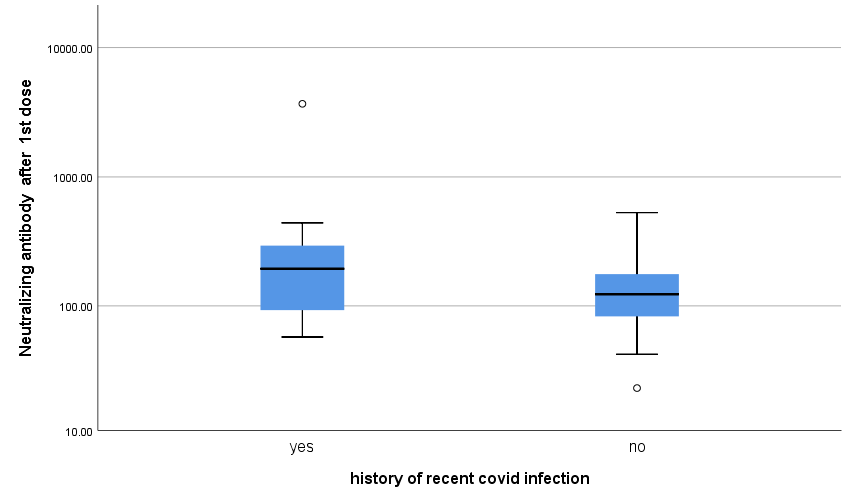


**Figure S1:** history of recent COVID-19 infection

**S2:** Demographic characteristics of included subjects

Table S1: Correlation between neutralizing antibody results on vaccination with AstraZeneca, and D-dimer and PLT

|  | | **Asterazeneka** | | |
| --- | --- | --- | --- | --- |
|  |  | **Neutralizing antibody Before vaccination** | **Neutralizing antibody after 1st dose** | **Neutralizing antibody after 2nd dose by 6 months** |
| **PLT before 1st dose** | **Correlation Coefficient** | 0.252 | 0.154 | 0.150 |
|  | **P value** | 0.021 | 0.161 | 0.175 |
|  | **N** | 84 | 84 | 84 |
| **PLT after 1st dose by 5 days** | **Correlation Coefficient** | 0.299 | 0.152 | 0.162 |
|  | **P value** | 0.006 | 0.167 | 0.140 |
|  | **N** | 84 | 84 | 84 |
| **D-dimer before the 1st dose** | **Correlation Coefficient** | 0.010 | 0.100 | 0.021 |
|  | **P value** | 0.928 | 0.363 | 0.847 |
|  | **N** | 84 | 84 | 84 |
| **D-dimer after the 1st dose by 5 days** | **Correlation Coefficient** | -0.216- | -0.028- | -0.036- |
|  | **P value** | 0.048 | 0.799 | 0.748 |
|  | **N** | 84 | 84 | 84 |
| **D-Dimer after 6 months** | **Correlation Coefficient** | -0.148- | -0.033- | 0.142 |
|  | **P value** | 0.179 | 0.763 | 0.197 |
|  | **N** | 84 | 84 | 84 |


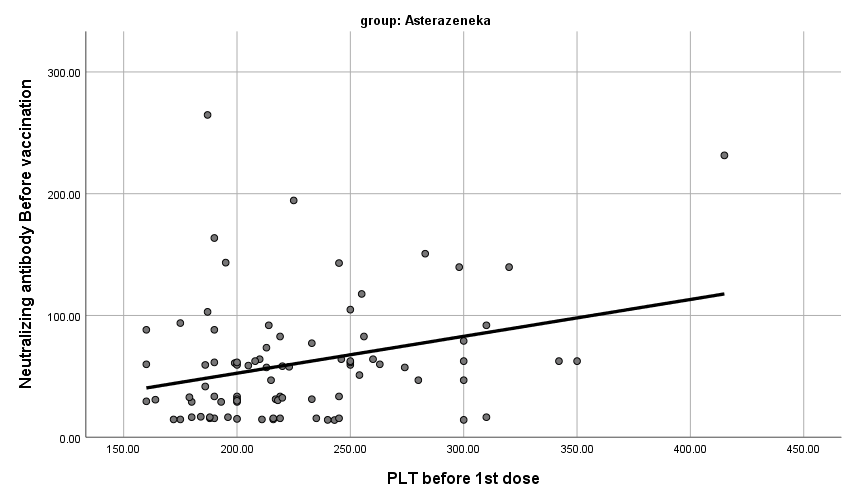


**Figure S3:** Correlation between NAbs before the 1^st^ dose of vaccination with AstraZeneca


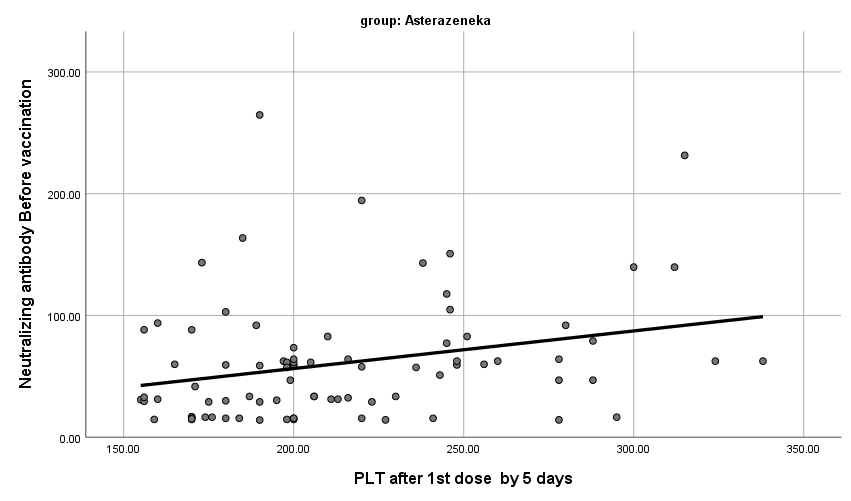


**Figure S4:** Correlation between NAbs following the 1^st^ dose of vaccination with AstraZeneca


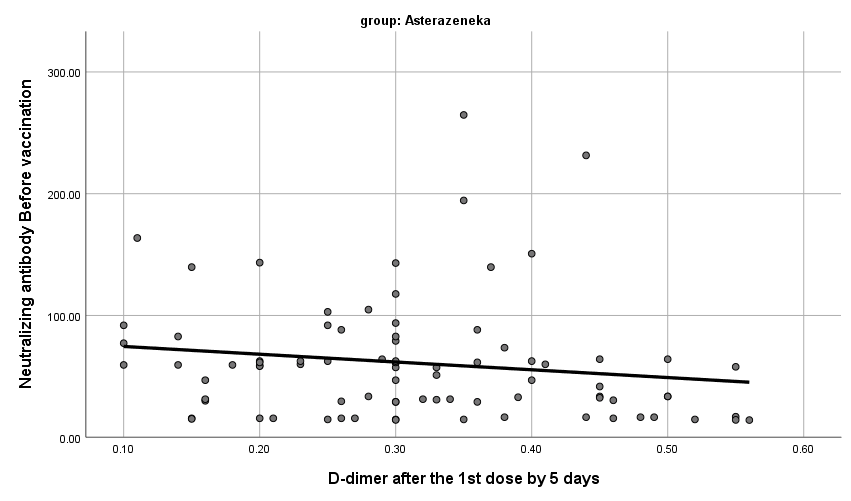


**Figure S5:** Correlation between NAbs following vaccination with AstraZeneca after the 1^st^ dose by 5 days

Table S2: Correlation between neutralizing antibody results before and after vaccination with Sinovac and D-dimer and PLT

|  | | **Sinovac** | | |
| --- | --- | --- | --- | --- |
|  |  | **Neutralizing antibody Before vaccination** | **Neutralizing antibody after 1st dose** | **Neutralizing antibody after 2nd dose by 6 months** |
| **PLT before 1st dose** | **Correlation Coefficient** | 0.167 | -0.190- | -0.250- |
|  | **P value** | 0.445 | 0.385 | 0.251 |
| **PLT after 1st dose by 5 days** | **Correlation Coefficient** | 0.113 | -0.217- | -0.180- |
|  | **P value** | 0.607 | 0.320 | 0.411 |
| **D-dimer before the 1st dose** | **Correlation Coefficient** | -0.313- | -0.155- | -0.105- |
|  | **P value** | 0.145 | 0.481 | 0.633 |
| **D-dimer after the 1st dose by 5 days** | **Correlation Coefficient** | -0.119- | -0.007- | -0.151- |
|  | **P value** | 0.589 | 0.975 | 0.491 |
| **D-Dimer after 6 months** | **Correlation Coefficient** | -0.107- | -0.145- | 0.082 |
|  | **P value** | 0.627 | 0.510 | 0.710 |

Table S3: Correlation between neutralizing antibody results before and after vaccination with mRNA vaccines (Moderna& Phizer) and D-dimer and PLT

|  | | **Moderan and Pfizer** | | |
| --- | --- | --- | --- | --- |
|  |  | **Neutralizing antibody Before vaccination** | **Neutralizing antibody after 1st dose** | **Neutralizing antibody after 2nd dose by 6 months** |
| **PLT before 1st dose** | **Correlation Coefficient** | 0.051 | -0.163- | -0.221- |
|  | **P value** | 0.784 | 0.382 | 0.231 |
| **PLT after 1st dose by 5 days** | **Correlation Coefficient** | 0.131 | -0.137- | -0.240- |
|  | **P value** | 0.484 | 0.464 | 0.194 |
| **D-dimer before the 1st dose** | **Correlation Coefficient** | 0.023 | 0.202 | 0.106 |
|  | **P value** | 0.904 | 0.277 | 0.570 |
| **D-dimer after the 1st dose by 5 days** | **Correlation Coefficient** | 0.054 | 0.214 | 0.211 |
|  | **P value** | 0.773 | 0.249 | 0.254 |
| **D-Dimer after 6 months** | **Correlation Coefficient** | 0.615 | 0.238 | 0.174 |
|  | **P value** | <0.001 | 0.197 | 0.349 |


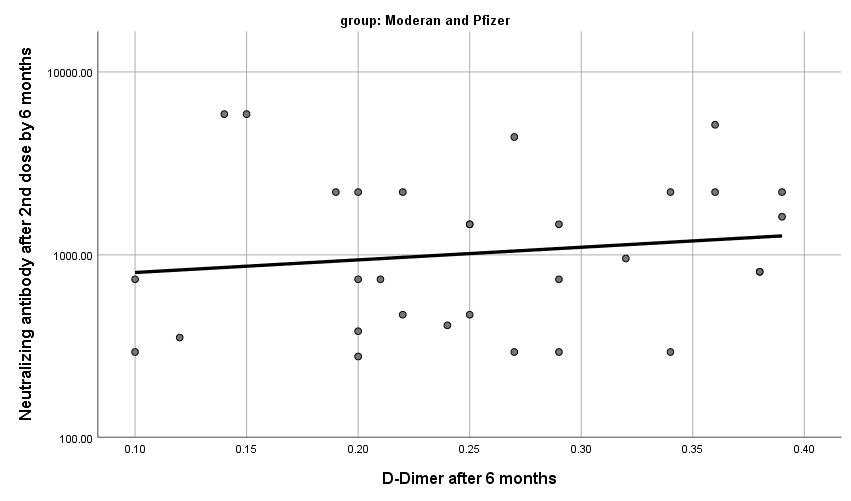


**Figure S6:** Correlation between NAbs following vaccination with Moderna & Pfizer after the 6 months of the second dose

Table S4: Correlation between age and the NAbs response before and after vaccination with different vaccines

| **Age** |  | **NAbs before vaccination** | **NAbs after 1^st^ dose** | **NAbs after 2^nd^ dose** |
| --- | --- | --- | --- | --- |
| **AstraZenica** | **Correlation coefficient** | -0.359- | -0.226- | -0.078- |
|  | ***p-*value** | 0.001 | 0.039 | 0.482 |
| **Sinovac** | **Correlation coefficient** | -0.245- | -0.089- | -0.166- |
|  | ***p-value*** | 0.260 | 0.686 | 0.450 |
| **Moderna&Pfizer** | **Correlation coefficient** | -0.385- | -0.334- | -0.219- |
|  | ***p-value*** | 0.033 | 0.066 | 0.237 |

Data are presented as median (percentiles).

Table S5: NAbs level in male and female HCWs before and after vaccination with different types of COVID-19 vaccines

|  |  | **Male** | **Female** | **p-value** |
| --- | --- | --- | --- | --- |
| **AstraZeneca** | **Before vaccination** | 57.55 (14.25-264.71) | 57.81(14.07-231.42) | 0.811 |
|  | **After 1^st^ dose** | 120.40 (44.79-342.59) | 117.6(41.67-529.41) | 0.782 |
|  | **After 2^nd^ dose** | 294.12(88.24-470.59) | 294.12(119.4-500) | 0.312 |
| **Sinovac** | **Before vaccination** | 41.67(34.15-57.29) | 46.09(14.6-222.21) | 1.000 |
|  | **After 1^st^ dose** | 82.72 (82.72-136.03) | 110.2 (22.4-294.14) | 1.000 |
|  | **After 2^nd^ dose** | 296.3 (231.4-411.7) | 305.5 (80.8-529.4) | 0.966 |
| **Moderna&**  **Pfizer** | **Before vaccination** | 57.29 (57.29-57.29) | 59.38 (24.78-500) | 0.774 |
|  | **After 1^st^ dose** | 294.1(294.1-294.1) | 294.12(73.53-3675) | 0.839 |
|  | **After 2^nd^ dose** | 2205 (2205-2205) | 808.50(277.78-5880) | 0.452 |

Data are presented as median (percentiles)
